# Effects of Whole-Body Cryotherapy vs. Far-Infrared vs. Passive Modalities on Recovery from Exercise-Induced Muscle Damage in Highly-Trained Runners

**DOI:** 10.1371/journal.pone.0027749

**Published:** 2011-12-07

**Authors:** Christophe Hausswirth, Julien Louis, François Bieuzen, Hervé Pournot, Jean Fournier, Jean-Robert Filliard, Jeanick Brisswalter

**Affiliations:** 1 Research Department, National Institute of Sport, Expertise and Performance (INSEP), Paris, France; 2 Laboratory of Human Motricity, Education and Health, University of Nice Sophia-Antipolis, Nice, France; 3 Medical Department, National Institute of Sport, Expertise and Performance (INSEP), Paris, France; Universidad Europea de Madrid, Spain

## Abstract

Enhanced recovery following physical activity and exercise-induced muscle damage (EIMD) has become a priority for athletes. Consequently, a number of post-exercise recovery strategies are used, often without scientific evidence of their benefits. Within this framework, the purpose of this study was to test the efficacy of whole body cryotherapy (WBC), far infrared (FIR) or passive (PAS) modalities in hastening muscular recovery within the 48 hours after a simulated trail running race. In 3 non-adjoining weeks, 9 well-trained runners performed 3 repetitions of a simulated trail run on a motorized treadmill, designed to induce muscle damage. Immediately (post), post 24 h, and post 48 h after exercise, all participants tested three different recovery modalities (WBC, FIR, PAS) in a random order over the three separate weeks. Markers of muscle damage (maximal isometric muscle strength, plasma creatine kinase [CK] activity and perceived sensations [i.e. pain, tiredness, well-being]) were recorded before, immediately after (post), post 1 h, post 24 h, and post 48 h after exercise. In all testing sessions, the simulated 48 min trail run induced a similar, significant amount of muscle damage. Maximal muscle strength and perceived sensations were recovered after the first WBC session (post 1 h), while recovery took 24 h with FIR, and was not attained through the PAS recovery modality. No differences in plasma CK activity were recorded between conditions. Three WBC sessions performed within the 48 hours after a damaging running exercise accelerate recovery from EIMD to a greater extent than FIR or PAS modalities.

## Introduction

Endurance events such as long distance running, cycling or triathlon competitions require extensive physical and psychological involvement of the athlete during both the training and the competition in order to achieve success. Well-trained runners complete training sessions practically every day or two, and so acute recovery becomes a vital factor in supporting supplementary training loads or competitions [1], [2]. Especially after a running session, where a large proportion of eccentric work is performed, muscular recovery becomes pertinent. It is well documented that eccentric contractions, which involve force generation in a lengthening muscle, procure severe structural damage in muscles, affecting their contractile properties [3].Within days after exercise, these structural alterations are classically accompanied by physiological and subjective perceptions of muscle damage that delay recovery. The release of muscular enzymes (e.g. creatine kinase, CK) into the plasma and sensations of pain or discomfort (i.e. delayed-onset-muscle-soreness, DOMS) typically occur after eccentric loading of the skeletal muscle and are classically used to study the extent of muscle damage [4], [5], [6]. In addition, the ensuing decline in maximal force generating capacity constitutes a relevant indicator of exercise-induced muscle damage (EIMD) [7].

A variety of authorized strategies are proposed to alleviate the deleterious effects of EIMD and enhance recovery such as nutritional supplementation [7], post-exercise massages [8], compressive garments [5], water immersion [9], whole-body cryotherapy (WBC) [10] or body expositions to the warmth [11]. Therapies based on temperature diminution through cold water immersion (4°C to 16°C) or local application of cooling apparatus (i.e. ice-vests, cold towels or ice packs) are some of the most recent strategies for promoting recovery from endurance efforts [12], [13]. Whatever the technique used, the main beneficial effect of cold during recovery is the cold-related vasoconstriction that may limit vessels' permeability and thus inflammatory processes, reducing muscle pain [14]. Based upon this framework, in addition to the different cold methods currently available, the use of WBC to alleviate pain and inflammatory symptoms has recently been suggested [15]. WBC consists of exposure to very cold air that is maintained at −110°C to −140°C in a special temperature-controlled cryochamber, generally for 3 to 4 minutes [16]. Despite the increasing popularity of WBC in sports, very few studies have tried to verify its efficacy on recovery. Recently, Banfi et al. [17] have recorded beneficial effects of a one week course of daily sessions of WBC on recovery from EIMD in professional rugby players. Results indicate a significant reduction in muscular enzymes (creatine kinase [CK] and lactate dehydrogenase [LDH]) and pro-inflammatory cytokines into the plasma, associated with an increase in anti-inflammatory cytokines. Additionally, Pournot et al. [18] recently reported that a single exposure to WBC significantly alleviated inflammation after a strenuous exercise run mainly composed of eccentric contractions. According to these results, WBC could hasten muscular recovery after exercise by reducing inflammatory processes, vascular permeability and subsequent edema development [15]. In an attempt to better understand the effects of WBC on human physiology, additional studies have been recently conducted with healthy men. The main results indicate a number of physiological changes occurring after body exposures to cold. These changes principally concern hematologic values [19], immunological and inflammatory responses [20], [21], and antioxidant/prooxidant balance [20], [22]. As an example, Lubkowska et al. [20], [21] have reported an increase in body's immunity, associated with a decrease in total oxidative status and inflammatory response after repeated WBC sessions (10 to 20 sessions). Klimek et al. [23] have also shown an improvement in anaerobic capacity after 10 WBC sessions, principally explained by metabolic changes (i.e. increased activity of anaerobic glycolitic enzymes) and a better tolerance to pain, highlighted by an increase in blood lactate concentration. Practically, it seems that a sufficient number of WBC sessions (at least 10 sessions) are necessary to stimulate an immunological response, while antioxidant and anti-inflammatory effects would be obtained from the first session. Such reactions could be particularly beneficial during the recovery period following exercise, and reinforce the significance of WBC in a sporting context.

Other recovery modalities such as far-infrared (FIR) therapy are also used to relieve pain in patients with muscular disorders and more recently have been considered as an efficient recovery strategy in sport [24], [25]. FIR therapy generally consists of a 30 min body exposition to FIR in a specially built apparatus. FIR are not visible by the human eye but are experienced by the warmth it produce. Potential positive effects of FIR during recovery are mainly based on the increase of peripheral flow due to the warmth-related vasodilatation, which could enhance the evacuation of edema, limiting inflammation and perceived pain, and enhancing muscle repair [26]. Moreover, by penetrating into the skin, the FIR energy could break agglomerates of water molecules in smaller groupings, which could reduce edema and facilitate the release of metabolic wastes [26]. However, similarly to WBC, the effect of FIR on recovery is mainly based on observations and habitual utilization. To date, the only verified effect of FIR is a reduction of perceived muscle pain and tiredness, induced by an increase in endorphin production [25], [27].

In order to determine the most appropriate recovery strategy for running-induced muscle damage, this study compared three different recovery modalities (WBC, FIR and passive [PAS] recovery) on symptoms of EIMD following a strenuous simulated trail running race performed by highly-trained endurance runners.

## Materials and Methods

### Ethical standards

These experiments were conducted according to the Helsinki Declaration (1964: revised in 2001) and the protocol was approved by the local Ethics committee (Ile-de-France, XI, France. Ref. 200978). All subjects gave their written informed consent before the initiation of the experiment.

### Subjects

Nine well-trained runners participated in the study (see Table 1 for characteristics), all with similar training levels and statures. The criteria used for selection of runners were a minimal performance of 38 min on a 10 km running race, and a minimum of 4 training sessions per week over the last year before the experiment. Selected runners regularly engaged in long distance running events (e.g. marathon, trails) and presented no contraindications to WBC or FIR therapy, such as claustrophobia and cold/warmth hypersensitivity. All subjects were volunteers and were informed about the study protocol, the risks of tests and investigations, and their rights according to the Declaration of Helsinki. Participants gave their written informed consent and the study was approved by the local Ethics Committee (Île-de-France XI, France; Ref. 200978) before its initiation.

**Table 1:** Characteristics of runners.

Variables (units)	Subjects (n = 9)
Age (years)	31.8±6.5
Height (m)	1.79±0.06
Weight (kg)	70.6±6.5
Training in running (sessions.week^−1^)	4.8±1.3
VO_2_max (ml.min^−1^.kg^−1^)	62.0±3.9
MAS (km.h^−1^)	18.7±1.1
V VT1 (km.h^−1^)	14.2±0.7
V VT2 (km.h^−1^)	16.7±1.2
10 km personal best (hour:min:sec)	00:34:48±00:02:35
Semi-marathon personal best (hour:min:sec)	01:17:12±00:06:12
Marathon personal best (hour:min:sec)	02:45:38±00:15:58

Data are means ± SD.

Legend Table 1: VO_2_max (ml.min^−1^.kg.^−1^), maximal oxygen consumption; MAS, maximal aerobic speed; V VT1 velocity at 1^st^ ventilatory threshold; V VT2, velocity at 2^nd^ ventilatory threshold.

### Experimental design

This study was conducted in order to analyze the effect of three different recovery modalities on EIMD following a simulated trail running race. In three non-adjoining weeks, the nine runners performed three identical repetitions of a simulated trail run on a motorized treadmill, designed to induce muscle damage. Within the first hour (post), 24 h (post 24 h), and 48 h (post 48 h), after each strenuous running exercise, all participants tested one of the three recovery modalities (WBC, FIR, PAS) presented in a random order. Classical indicators of EIMD such as, plasma CK activity, isometric maximal voluntary torque, and perceived sensations of pain, tiredness and well-being (typically grouped under the term DOMS), were assessed immediately before (pre) and after (post) each simulated running trail, and after each of the three recovery sessions (post 1 h, post 24 h, post 48 h). All participants performed three identical running trails and used all the three recovery modalities over the experiment. Between trials, a minimum of three weeks of low intensity training was ensured, in order to allow a complete muscular recovery. However, in order to limit and control the development of additional EIMD, subjects were asked not to train for the three days preceding and succeeding the data recording.

One week before the experiment, subjects were familiarized with the test scheme and location and preliminary testing was performed. From this week onwards until the end of the experimentation period, the training loads of all subjects were controlled by asking them to train with a heart rate monitor, and they did not use other recovery strategies like stretching, nutritional supplementation, electro stimulation, or cold water immersion. Moreover, in order to control the influence of other recovery modalities, nutritional recommendations were sent to runners during all the experiment and they were asked to respect identical menus during the three days preceding and succeeding the running sessions.

### Preliminary testing

Maximal oxygen uptake (VO_2max_) was determined on a motorized treadmill (H/P/Cosmos® Saturn, Traunstein, Germany). The test consisted of a 6 min warm-up at 12 km.h^−1^ and an incremental period in which the running speed was increased by 1 km.h^−1^ every 2 min until volitional exhaustion. Oxygen uptake (VO_2_), minute ventilation (VE), and respiratory exchange ratio (RER) were continuously recorded with a breath by breath gas exchange analyzer (Quark CPET, Cosmed, Roma, Italy). Heart rate (HR) was recorded using a chest belt (Cosmed wireless HR monitor, Roma, Italy). The criteria used for the determination of VO_2max_ were threefold: a plateau in VO_2_ despite an increase in power output, a RER above 1.1, and a heart rate (HR) above 90% of the predicted maximal HR [28].VO_2max_ was determined as the average of the four highest VO_2_ values recorded (mean VO_2_max: 62.0±3.9 ml.min^−1^.kg^−1^). The first and the second ventilatory thresholds (VT1 and VT2) were determined as described by Wasserman et al. [29].The maximal aerobic speed (MAS) was the highest running velocity completed for 2 min (mean MAS: 18.7±1.1 km.h^−1^). After this preliminary running exercise, subjects were familiarized with the ergometer used to evaluate lower limb muscle strength and with the recovery apparatus.

### Simulated trail running race

**Figure 1:**
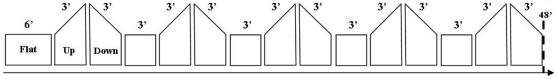
Schematic representation of the simulated running trail. Flat, 0% gradient section; Up, +10% gradient section; Down, −15% gradient section.

Once a month within a three months' period, subjects completed a simulated trail running race with a large amount of downhill sections (total downhill time: 15 min), well-known to induce muscle damage [30], [31], [32], on the same treadmill used for preliminary testing. The trail run was designed to replicate as completely as possible the race constraints encountered in a trail run. The race lasted 48 min and was divided in 5 blocks. The first block included 6 min on the flat (0% gradient), followed by 3 min uphill (+10% gradient) and 3 min downhill (−15% gradient). Velocity was continuously adjusted as a function of gradient in order to obtain a variety of intensities and elicit a similar metabolic demand to trail races in the field (Fig. 1). Therefore, velocity at 0% gradient was between VT1 and VT2 (mean Vflat: 15.5±0.9 km.h^−1^), while velocity at +10% gradient corresponded to ∼80% (mean Vuphill: 11.1±0.9 km.h^−1^) of the maximal aerobic velocity at this gradient [33], and velocity at −15% corresponded to velocity at VT1 (mean Vdownhill: 14.2±0.7 km.h^−1^). Blocks 2–5 consisted of 3 min at 0°, followed by 3 min uphill and 3 min downhill at the gradients and velocities previously described.

### Recovery interventions (WBC vs. FIR vs. PAS)

Subjects were randomly assigned to one recuperation modality (WBC, FIR or PAS) to be used after the simulated trail running race (post), post 24 h and post 48 h when EIMD are typically reported to be the most important [32], [34]. All subjects used each of the recovery modalities in the course of the experiment. WBC sessions were administered under medical supervision, in a specially built, temperature-controlled unit (Zimmer Elektromedizin, Germany), which consists of three rooms (−10, −60 and −110°C). The temperature of all rooms remained constant throughout the experiment. During each WBC session, subjects traversed the warmer rooms and remained in the therapy room for 3 min. In the familiarization session, exposure was reduced to 1 min. Subjects were instructed to dry eventual sweat, wear a bathing suit, surgical mask, earband, triple layer gloves, dry socks and sabots. During the 3 min, subjects avoided tension by slightly moving their arms and legs by walking. After the WBC session, subjects spent 10 min seated comfortably in a temperate room (24°C) wearing a bath robe, and were allowed to dress themselves as warmly as they wished to avoid a subjective sensation of cold. The second recovery modality was a 30 min exposure to far-infrared radiation (Inovo, IRL technology, Montpellier, France). Subjects lay in a supine position on the table of the apparatus, clothed only in a bathing suit and thus exposing the whole body, except for the head, to FIR (4–14 µm, 45°C).

Finally, the last recovery modality was a passive recovery (control modality) during which subjects were seated comfortably in an armchair for 30 min, located in the same temperate room previously presented.

### Data recording

#### Indicators of exercise-induced muscle damage

Indicators of EIMD included maximal muscle force, muscle enzyme creatine kinase (CK) activity in the plasma, and perceived sensations of muscle pain, tiredness and well-being, which have been commonly used as indirect markers of muscle damage in previous studies [5], [32], [35].All markers were measured in pre, post, post 1 h, post 24 h and post 48 h conditions.

#### Muscle torque assessment

Knee extensors' isometric maximal voluntary torque was assessed at a 70° knee angle with an isokinetic ergometer (Con-Trex Multi-Joint System, Dübendorf, Switzerland).After a brief warm-up which consisted of 5 min low intensity running and submaximal isometric contractions, subjects were placed in a seated position in the ergometer chair with their hips and thigh strapped to the seat. Subjects were instructed to extend their knee “as fast and as hard as possible” [36] and each maximal contraction was sustained for 5 s. Three isometric maximal voluntary contractions (MVC) of the knee extensor muscles were performed with rest periods of 60 s in between. Maximal MVC performance was defined as the highest peak torque value of the three maximal attempts.

#### Plasma creatine kinase activity

Each time, blood samples were collected before MVC had been performed, in order to avoid a potential influence of this maximal exercise on CK level into the plasma. Plasma CK activity was determined from a 5 ml sample of whole blood collected into vacutainer tubes via antecubital venipuncture. Once the blood sample was taken, tubes were mixed by turning and placed on ice for 30 s before centrifugation (10 min, 3000 rev.min^−1^, 4°C). The obtained plasma sample was then stored in multiple aliquots (Ependorf type, 500 µl per samples) at −80°C until analysis. As a marker of sarcolemma disruption, plasma CK activity was measured spectrophotometrically by using commercially available reagents (Roche/Hitachi, Meylan, France).

#### Perceived sensations

The effects of recovery interventions on EIMD were also recorded through the assessment of the perceived sensations of subjects. The Mindeval system (www.mindeval.com) was used to collect the data (Mindeval GydleInc. Québec, CANADA). This system is comprised of a web interface with a database and a stand-alone application. In Pre, Post, Post 1 h, Post 24 h, and Post 48 h conditions, participants entered their personal key and answered three areas of questions related to 1) pain, 2) tiredness, and 3) well being. For example, to answer the question “how sore are you?” subjects use the computer mouse to move the indicator between the two ends “no pain” and “maximum pain”. The software records the location of the indicator with a number ranging between 0 (no pain) and 100 (maximum pain). The collected data was stored on a secured server. Before the initiation of the study, subjects were accustomed to the software, and the questions relative to their subjective sensations were thoroughly explained to be sure that all subjects understood the same meaning.

### Statistical analysis

All data were expressed as mean ± standard deviation (SD). A two-way analysis of variance (recovery modality×period) for repeated measures was performed to analyze the effects of the running trail (Pre vs. Post, Post 1 h, Post 24 h, Post 48 h) and recovery intervention (Post vs. Post 1 h, Post 24 h, Post 48 h) with MVC, plasma CK activity, and perceived sensations as dependent variables. The LSD Fischer post-hoc test was used to determine the between-means differences if the analysis of variance revealed a significant main effect for period or interaction of recovery modality×period. For all statistical analyses, a *p*<0.05 value was accepted as the level of significance.

## Results

No significant differences between running sessions were observed in absolute terms at baseline for maximal voluntary torque, plasma CK activity, and perceived sensations.

### Maximal voluntary contractions

**Figure 2:**
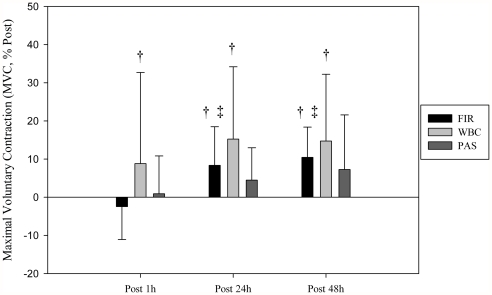
Recovery of knee extensor's maximal voluntary contraction (MVC, % of post), assessed after each of the three recovery sessions (post 1 h, post 24 h, post 48 h). † significantly different from post condition (*p*<0.05), ‡ significantly different from post 1 h condition (*p*<0.05).

Results indicated a significant MVC decline immediately after the trail run whatever the groups, without differences between them (mean post MVC decline for all subjects and sessions: −9.6%, *p*<0.05). MVC capacity was recovered after the first WBC session (post 1 h), while it was recovered later with FIR (post 24 h), and did not recover in the PAS condition (Fig. 2).

### Plasma creatine kinase activity

In all subjects, CK activity significantly increased after the simulated trail running race (post: +51.7%, *p*<0.05). No effect of recovery modalities on CK activity was recorded in all testing periods (post 1 h, post 24 h, and post 48 h). The peak of CK activity following exercise occurred 24 h post-exercise (post 24 h) without significant differences between recovery conditions (Table 2).

**Table 2:** Indicators of exercise-induced muscle damage (EIMD), assessed before (pre) and after (post) the simulated running trail, and after the three recovery sessions (post 1 h, post 24 h, post 48 h).

Variables (units)	Pre	Post	Post 1 h	Post 24 h	Post 48 h
**CK (% of pre)**					
**FIR**	0.0±0.0	40.5±18.4*	44.2±20.9*	192.3±179.3* † ‡	107.5±121.1* $
**WBC**	0.0±0.0	58.2±18.9*	73.9±33.4*	318.9±224.7* † ‡	195.3±141.6* $
**PAS**	0.0±0.0	56.4±25.1*	63.7±26.5*	231.8±132.1* † ‡	137.6±99.8* $
**Pain (/100)**					
**FIR**	1.6±3.2	61.9±19.0*	58.3±18.4*	49.3±29.1*	45.2±29.1* †
**WBC**	0.2±0.7	60.6±20.7*	31.7±23.8* †	33.3±26.1* †	39.0±24.0* †
**PAS**	0.1±0.3	55.7±18.2*	44.3±23.7*	53.9±25.5*	58.9±19.0*
**Tiredness (/100)**					
**FIR**	8.3±9.8	75.3±11.2*	67.8±21.3*	65.8±20.0*	61.8±15.9*
**WBC**	5.2±9.8	77.9±13.3*	44.6±26.3* †	35.9±19.4* †	46.6±24.0* †
**PAS**	8.7±12.3	65.4±26.6*	52.2±27.0*	49.2±21.4*	60.7±26.7*
**Well-being (/100)**					
**FIR**	86.8±16.9	56.6±31.9*	67.9±28.2*	66.9±27.6*	72.4±19.2†
**WBC**	77.7±25.2	65.4±26.6	74.9±26.7	87.1±0.0†	81.2±20.4†
**PAS**	93.9±9.0	58.4±26.8*	69.8±25.3*	65.4±21.1*	68.7±28.1*

Data are means ± SD.

Legend Table 2: CK, plasmatic creatine kinase activity; FIR, far infrared; WBC, whole body cryotherapy; PAS, passive.

*significantly different from pre condition (*p*<0.05);

† significantly different from post condition (*p*<0.05);

‡ significantly different from post 1 h condition (*p*<0.05);

$ significantly different from post 24 h condition (*p*<0.05).

### Perceived sensations

Psychological parameters were influenced both by the strenuous running exercise and recovery modality during the following 48 h (Table 2). In all subjects, the perceived pain and tiredness significantly increased immediately after exercise (Post) and remained elevated post 1 h, post 24 h, and post 48 h. Pain and tiredness were reduced after the first WBC session (post 1 h), whereas FIR only reduced pain at a later point in time (post 48 h). Neither pain nor perceived tiredness was modified by passive recovery during the 48 h after exercise. In FIR and PAS conditions, well-being was altered after the running trail and remained lower than pre-exercise values in all testing periods excepted in post 48 h in the FIR condition. Well-being was, however, higher than post exercise values at post 24 h in the WBC condition and post 48 h in the FIR condition.

## Discussion

This study was designed to compare the effects of different recovery strategies following a damaging simulated trail run, performed by highly-trained endurance runners. As expected, this running exercise induced significant muscle damage, manifested through a reduction in maximal torque generating capacity, an increase in plasma CK activity, and an increase in pain and tiredness sensations. The main results are that MVC and perceived sensations were recovered after the first WBC session (post 1 h) while recovery took 24 hours in the FIR recovery modality and was not achieved with the PAS modality. However, no beneficial effect of recovery modality was observed on plasma CK activity.

A decrease in maximal torque generating capacity is widely accepted as a marker of muscle damage following a strenuous exercise. This decline is magnified when exercise involves eccentric contractions [30], [37]. In the present study, the mean MVC decrease for all subjects over the three running trails was −9.6, −8.2, −2, and −0.8% respectively post, post1 h, post 24 h, and post 48 h after the simulated running race. This MVC decline is less accentuated than in previous field studies on longer races (marathon or trail running races), where −16 to −37% MVC declines were recorded [7], [38], [39]. Moreover, it could be supposed that simulated running races on treadmill are classically less damaging for muscles than real running races where the courses are often more difficult, with bigger variations in level, unstable surfaces, and variations in atmospheric conditions. Increases in pain, tiredness, and plasma CK activity (mean peak in post 24 h: +247%) confirm the success of the present protocol in generating muscle damage, but still in lower proportions than longer overground running protocols [38], [40].

The most beneficial effects of recovery sessions organized within the first 48 hours after the simulated trail running race were recorded with the WBC modality. MVC was recovered after the first WBC session (post 1 h), while recovery took 24 h with FIR, and was not attained through PAS recovery. On contrary, Costello et al. [41] reported no beneficial effect of 2 WBC sessions on the recovery of maximal muscle strength after repeated eccentric contractions of knee extensors. However, in the study of Costello et al. [41], participants were not highly-trained runners as in our study, and therefore were not accustomed to eccentric contractions, which may explain this absence of positive effect of WBC. Additionally, multiple studies on the effects of cold therapy through ice or water immersion on maximal force recovery present contradictory results depending on the activity and its intensities [9], [13], [14], [42], [43]. However, the majority of studies suggest that short-term whole body immersion is beneficial to restoring force-generating capacity and repeating endurance performance when performed immediately after exercise [13], [44].The main hypothesis suggested to explain this effect is related to the fall in core temperature during the cold exposition inducing, via a vasoconstriction mechanism, a decrease in vessels permeability to immune cells, and thus reducing the edema and inflammatory process and/or pain [14]. However, long-term cold immersion could be detrimental for recovery by inducing an increase in TNF-α (i.e. a proinflammatory cytokine), lymphocytes and monocytes [45], [46]. According to these authors, this immunostimulating effect of cold could be related to an enhanced noradrenalin response to the cold [46]. In another side, published data suggest that repeated short-term expositions to WBC have beneficial effects by reducing inflammatory processes, and having a mobilization effect on the immunological system. First, Banfi et al. [17] have reported no effect of 3 min WBC on immunological parameters in rugby players after 5 days of WBC, but a decrease in pro- inflammatory cytokines associated with an increase in anti-inflammatory cytokines. More recently, Lubkowska et al. [20], [21] have reported a significant increase in while blood cell count after numerous (10 to 20 sessions) WBC expositions in healthy men, systematically accompanied with an increase in anti-inflammatory cytokines. A significant anti-inflammatory effect was also reported by Pournot el al. [18] after a single WBC session performed after an exhaustive run in well-trained runners. Both the anti-inflammatory and pro-inflammatory cytokines were positively modified after the WBC session, mainly explained by a vasoconstriction mechanism at muscular level. According to Miller et al. [22], 10 WBC sessions could also significantly stimulate the antioxidant protection by increasing the amount of enzymatic and non-enzymatic antioxidant species. Changes in while blood cell count, in pro- and anti-inflammatory cytokines, and changes in both the total oxidative and antioxidative status, after WBC exposures confirm the significance of WBC to improve body's defenses and thus post-exercise recovery. Moreover, a cold-related reduction in nervous activity, combined with an increased endorphin concentration could have an analgesic effect, reducing the perception of fatigue and pain [34], [47], and allowing subjects to develop more force. The perception of pain or tiredness is determined by both physiological and psychological influences, and thus constitutes a relevant indicator of muscle recovery to support physiological findings [48]. Similar to MVC capacity, the present study recorded beneficial effects of WBC on psychological recovery within days after exercise. Pain and tiredness sensations subsequent to the simulated trail running race were reduced after the first WBC session, while pain sensation only was lowered later (post 48 h) by using FIR, and no effect of PAS recovery was recorded. Beneficial effects of the WBC and FIR recovery modalities on well-being were also recorded post 24 h with WBC and post 48 h with FIR. All previous studies on cold exposure methods (i.e. ice application, cold water immersion) have indicated minimal or no effect of cold on recovery of psychological feelings after exercise [4]. Moreover, in the recent study of Costello et al. [41] muscle soreness sensations were not lowered after 2 WBC. As previously mentioned, it can be hypothesized that muscle damage could be higher in the study of Costello et al. [41] when compared with ours, mainly related to the fact that participants were not accustomed to eccentric contractions. Indeed, numerous studies have shown that the amount of EIMD can be dramatically different according to the training status, and that only one previous exposure to eccentric contractions provides a protective effect for muscle structures [49], [50]. Within this frame, our results seem to confirm a previous study conducted in the medical domain, in which WBC induced a reduction of depressive symptoms by enhancing well-being, sleep and relaxation [51], [52]. The results indicate that sufficiently low temperatures and a whole body exposure to cold seem to be beneficial in enhancing the sensation of recovery.

In contrast to previous studies, the WBC session did not influence plasma CK activity within the first 48 hours after exercise [15], [53], [54]. Indeed, in our study, no differences in absolute plasma CK concentrations were recorded before and after exercise between recovery modalities in all testing sessions. One hypothesis to explain the lack of effect of recovery modalities on CK concentrations could be the number of WBC recovery sessions performed after exercise. Banfi et al. [53] reported a significant −40% reduction in CK level after five days of daily WBC sessions in rugby players, and Wozniak et al. [54] reported a −34% decline in CK concentration after 10 WBC sessions performed by 21 kayakers before each training session. The repeated cold-related stimulations of noradrenalin could partly explain the decrease in CK concentration after WBC sessions, associated with a decline in prostaglandin PGE2 (i.e. an inflammatory mediator and vasodilator) which could reduce vascular permeability [53]. In contrast, our results show that 3 WBC sessions doesn't limit the increase in plasma CK activity generally related to structural damage of the muscle fibers. According to these results, it seems that repeated expositions (a minimum of 5 to 10 sessions) to WBC are required to stimulate recovery from muscle fiber damage by reducing muscle membrane breakdown or increased cell permeability induced by physical exercise.

In conclusion, this study was designed to compare the effects of three different recovery modalities (WBC vs. FIR vs. FIR) during the acute recovery period (post 48 h) following a damaging simulated trail run. WBC (3 min at −110°C) was the best recovery modality to hasten recovery from EIMD by limiting the torque loss and subjective sensations of pain, classically recorded after repeated eccentric contractions.
